# Prevention and Treatment of Venous Thromboembolism with New Oral Anticoagulants: A Practical Update for Clinicians

**DOI:** 10.1155/2013/183616

**Published:** 2013-02-21

**Authors:** Nay Min Tun, Thein Hlaing Oo

**Affiliations:** ^1^Division of Hematology and Oncology, The Brooklyn Hospital Center, 121 Dekalb Avenue, Brooklyn, NY 11201, USA; ^2^Section of Thrombosis and Benign Hematology, The University of Texas MD Anderson Cancer Center, Unit 1464, 1515 Holcombe Boulevard, Houston, TX 77030, USA

## Abstract

Traditional anticoagulants, such as warfarin and enoxaparin, have several limitations, including parenteral administration, need for laboratory monitoring, and ongoing dose adjustment, which may limit optimal patient care. Newer oral anticoagulants, such as direct thrombin inhibitors (e.g., dabigatran etexilate) and direct factor Xa inhibitors (e.g., rivaroxaban, apixaban, and edoxaban), have been developed to overcome these drawbacks, and thereby improve patient care. Several of these agents have been approved for use in the prevention and treatment of venous and/or systemic thromboembolism. The objective of this paper is to provide an overview of the available clinical trial data for these new oral anticoagulants in the prevention and treatment of venous thromboembolism and a practical update for clinicians.

## 1. Introduction

Venous thromboembolism (VTE) comprises deep vein thrombosis (DVT) and pulmonary embolism (PE). Although the exact incidence of VTE is not known, it is estimated to affect 900,000 patients each year in the United States [1]. Approximately one-third of these cases are fatal pulmonary emboli, and the remaining two-thirds are nonfatal episodes of symptomatic DVT or PE [1]. VTE is the second most common cause of extended hospital stay and the third most common cause of in-hospital mortality [2]. Because it causes considerable morbidity and mortality, VTE places a substantial burden on healthcare resources [3, 4]. 

Without thromboprophylaxis, the incidence of hospital-acquired DVT based on objective diagnostic screening is 10–40% among medical or general surgical patients and 40–60% among patients who have undergone major orthopedic surgery such as total knee replacement (TKR), total hip replacement (THR), and hip fracture surgery [5]. Patients with cancer are at a greater risk of new or recurrent VTE than patients without cancer. VTE risk is 3- to 5-fold higher in cancer patients who are undergoing surgery and 6.5-fold higher in cancer patients receiving chemotherapy than in patients who do not have cancer [6, 7]. 

**Figure 1:**
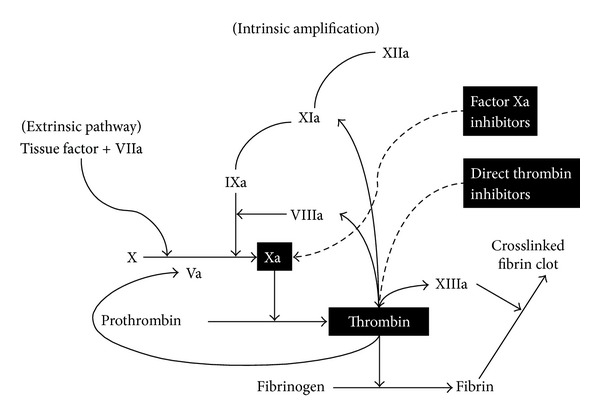
Site of action of new oral anticoagulants in the coagulation cascade.

The efficacy of traditional anticoagulants in preventing VTE in patients undergoing major orthopedic surgery and in hospitalized acutely ill medical patients is well established [5, 8–11]. However, these agents have several limitations that may limit optimal patient care, such as their parenteral administration, need for laboratory monitoring, and ongoing dose adjustment (Table 1) [12–16]. Newer oral anticoagulants, such as direct thrombin inhibitors (e.g., dabigatran etexilate) and direct factor Xa inhibitors (e.g., rivaroxaban, apixaban, and edoxaban), have been developed to overcome these drawbacks, and thereby improve patient care. Their pharmacologic targets in the coagulation cascade are described in Figure 1, and their general pharmacologic characteristics are summarized in Table 2. The objective of this paper is to provide an overview of the available clinical trial data for these new oral anticoagulants from the perspective of prevention and treatment of VTE and to provide a practical update for clinicians. 

## 2. Direct Thrombin Inhibitors

Thrombin is the final mediator in the coagulation cascade that facilitates the conversion of fibrinogen to fibrin (Figure 1). Thrombin also activates factor V, factor VIII, and platelet-bound factor XI, which generate additional thrombin [17]. Moreover, thrombin is a potent activator of platelets [17, 18]. Direct thrombin inhibitors inactivate fibrin-bound thrombin, which is an important trigger of thrombus expansion, and also directly inactivate free thrombin [19].

### 2.1. Dabigatran Etexilate

#### 2.1.1. Pharmacology

Dabigatran is a potent, competitive, reversible, thrombin inhibitor that binds directly to the active binding site of free or fibrin-bound thrombin in a concentration-dependent manner [20, 21]. After oral administration, dabigatran etexilate is absorbed via the gastrointestinal tract and rapidly hydrolyzed by nonspecific esterases in the gut, plasma, and liver to its active form, dabigatran [21]. Peak plasma concentration is achieved 0.5–2 hours after administration of the drug [22]. It has a half-life of 12–17 hours [20], an absolute bioavailability of 3–7%, and approximately 35% plasma protein binding [23]. Approximately 80% of dabigatran is excreted by the kidneys [24]. Dabigatran etexilate, but not dabigatran, is a substrate of P-glycoprotein (P-gp), an intestinal drug transporter, and its absorption is influenced by a number of P-gp inhibitors and inducers. Neither dabigatran etexilate nor dabigatran is metabolized by the cytochrome P450 system. In addition, dabigatran does not seem to inhibit or induce cytochrome P450 enzyme activity. Dabigatran induces dose-proportional and near-linear increases in activated partial thromboplastin time (aPTT), prothrombin time (PT), thrombin time (TT), and ecarin clotting time (ECT) [20].

#### 2.1.2. Primary VTE Prevention after Major Orthopedic Surgery

The use of dabigatran etexilate as a VTE prophylactic agent in major orthopedic surgery was evaluated in four phase III randomized, double-blind, noninferiority studies, the RE-MOBILIZE, REMODEL, RE-NOVATE, and RE-NOVATE II trials. The RE-MOBILIZE study indicated that over 12–15 days of treatment, dabigatran etexilate (150 mg or 220 mg once daily) was not as effective as enoxaparin (30 mg twice daily) in preventing total VTE and all-cause mortality in patients undergoing TKR [25]. However, the REMODEL study demonstrated that over 6–10 days of treatment, dabigatran etexilate (220 mg or 150 mg once daily) was noninferior to enoxaparin (40 mg once daily) for the prevention of VTE in patients undergoing TKR [26]. There was no significant difference in the frequency of major bleeding or overall rate of adverse events between either dose of dabigatran etexilate and enoxaparin. Similar findings were reported by the RE-NOVATE trial in patients who underwent THR and used extended VTE prophylaxis (for 28–35 days) [27]. The RE-NOVATE II trial again demonstrated that extended prophylaxis with oral dabigatran etexilate (220 mg once daily) was as effective as subcutaneous enoxaparin (40 mg once daily) in reducing the risk of VTE after THR, and superior to enoxaparin in reducing the risk of major VTE, with similar safety profiles [28].

Friedman et al. performed a pooled analysis of the RE-MOBLIZE, RE-MODEL, and RE-NOVATE trials and concluded that oral dabigatran etexilate doses of 150 mg or 220 mg daily were each as effective as 40 mg enoxaparin given subcutaneously once daily or 30 mg of enoxaparin given subcutaneously twice daily in reducing the risk of major VTE and VTE-related mortality after hip or knee replacement with a similar bleeding profile [29]. A similar pooled analysis by Huisman et al. of the RE-MOBILIZE, RE-MODEL, and RE-NOVATE studies demonstrated similar results [30]. Wolowacz et al. performed a meta-analysis of the RE-MODEL, RE-NOVATE, and RE-MOBILIZE trials and found no significant differences between the treatments with respect to VTE and bleeding endpoints [31].

#### 2.1.3. Treatment and Secondary Prevention of VTE

The RE-COVER study investigated the efficacy and safety of dabigatran etexilate (150 mg twice daily) versus warfarin (target international normalized ratio (INR) 2-3) in the treatment of acute VTE for 6 months. This randomized, double-blind, noninferiority trial reported that a fixed dose of dabigatran etexilate was as effective as warfarin in the treatment of VTE (hazard ratio with dabigatran etexilate for recurrent VTE, 1.10; 95% CI, 0.65–1.84) and had a safety profile similar to that of warfarin [32].

Two other phase III randomized double-blind clinical trials assessed the efficacy and safety of dabigatran etexilate for the extended treatment of VTE. In the RESONATE study, dabigatran etexilate (150 mg twice daily) was compared to placebo for an additional 6 months in patients who completed 6–18 months of treatment with a vitamin K antagonist [33]. Compared with placebo, dabigatran etexilate had a relative risk reduction of 92% for recurrent VTE without an increase in major bleeding events, although the incidence of clinically relevant bleeding was more frequent in the dabigatran etexilate group. In the REMEDY study, patients with VTE who had completed 3–12 months of anticoagulant therapy were randomized to treatment with dabigatran etexilate (150 mg twice daily) or warfarin (target INR 2-3) for an additional 6–36 months to prevent recurrent VTE and VTE-related death [34]. The study revealed that recurrent VTE events were more frequent in the dabigatran etexilate group than in the warfarin group (1.8% versus 1.3%; hazard ratio 1.44; *P* = 0.03). However, dabigatran etexilate was associated with a lower risk for bleeding than warfarin was (hazard ratio 0.71; 95% CI, 0.61–0.83). On the other hand, the incidence of acute coronary events in the dabigatran etexilate group was significantly higher than that in the warfarin group (0.9% versus 0.2%; *P* = 0.02).

#### 2.1.4. Practical Information

Dabigatran etexilate is currently approved in Europe and Canada for the prevention of VTE in patients undergoing hip or knee replacement [23, 35, 36]. However, it is not indicated for the treatment or secondary prevention of VTE (Table 4).

For the prevention of VTE after TKR, dabigatran etexilate at a dose of 110 mg should be given within 1–4 hours of the completion of surgery, and then continued at a dose of 220 mg once daily for 10 days [35]. For the prevention of VTE after THR, a similar dosing of dabigatran etexilate should be given for 28–35 days [35]. For both types of surgery, treatment with dabigatran etexilate should be delayed if patients are not hemodynamically stable or if hemostasis cannot be achieved within the first day after surgery. If dabigatran etexilate is delayed beyond the first day of surgery, the starting dose should be 220 mg orally once daily. 

Treatment with dabigatran etexilate is contraindicated in patients with a creatinine clearance (CrCl) < 30 mL/min [37]. For patients with a CrCl of 30–50 mL/min, the recommended dose is 75 mg given 1–4 hours after surgery and 150 mg daily thereafter [37]. In patients >75 years of age, the recommended dose for VTE prevention is similar to that of patients with a CrCl of 30–50 mL/min [37]. The use of dabigatran etexilate is not recommended in patients who have postoperative indwelling epidural catheters. In these patients, administration of the first dose of dabigatran etexilate should be delayed until at least 2 hours after the catheter is removed [37]. 

Dabigatran etexilate can be kept for 4 months once the bottle is opened. Patients should be advised to not store dabigatran etexilate in any other containers, such as pill organizers, and pharmacists should always dispense dabigatran etexilate in the original bottle in order to protect from moisture [23, 37]. Dabigatran etexilate is not currently approved for the treatment of VTE. 

## 3. Factor Xa Inhibitors

Factor Xa is the gatekeeper at the convergence of the intrinsic and extrinsic coagulation pathways (Figure 1). It facilitates the conversion of prothrombin to thrombin, which leads to the generation of over 1000 times more thrombin molecules owing to the amplification nature of the coagulation cascade [38]. Because it is the primary and rate-limiting source of amplification in the coagulation cascade, factor Xa is an attractive target for anticoagulant treatment.

### 3.1. Rivaroxaban

#### 3.1.1. Pharmacology

Rivaroxaban is a potent oral anticoagulant that directly and selectively inhibits free and clot-bound factor Xa. The drug demonstrates stable, dose-dependent, predictable pharmacokinetics with a fast onset-offset of action. The maximum concentrations of rivaroxaban occur 2–4 hours after oral intake. It has variable absolute oral bioavailability depending on the dose (80–100% for a 10 mg dose and 66% for a 20 mg dose), with a half-life of 5–9 hours in healthy subjects and 11–13 hours in elderly subjects [39–42]. Rivaroxaban exhibits a plasma protein binding percentage of approximately 92–95% and is metabolism via CYP3A4/5- and CYP2J2-dependent mechanisms [42]. Thirty percent of the drug is renally excreted as inactive metabolites, whereas 30–40% of the drug is renally excreted and the remainder is excreted in the feces as unchanged drug [43, 44]. Because the intestinal excretion appears to be mediated by P-gp, potent P-gp inhibitors may increase the drug concentrations [45]. Rivaroxaban does not inhibit other serine proteases such as trypsin [46]. 

#### 3.1.2. Primary VTE Prevention in Patients after Major Orthopedic Surgery

Use of rivaroxaban as a VTE prophylactic agent in major orthopedic surgery was evaluated in four phase III randomized, double-blind trials: the RECORD1 and RECORD2 trials, which evaluated the drug's use following THR, and the RECORD3 and RECORD4 trials, which evaluated the drug's use following TKR [47–50]. The RECORD1 study compared rivaroxaban (10 mg once daily for 35 days) to enoxaparin (40 mg once daily for 35 days) [47]. The RECORD2 study compared rivaroxaban (10 mg once daily for 31–39 days) with enoxaparin (40 mg once daily for 10–14 days followed by placebo) [48]. The RECORD3 study compared rivaroxaban (10 mg once daily for 10–14 days) with enoxaparin (40 mg once daily for 10–14 days), and the RECORD4 study compared rivaroxaban (10 mg once daily for 10–14 days) with enoxaparin (30 mg twice daily for 10–14 days) [49, 50]. In all four trials, rivaroxaban was reported to be superior to enoxaparin in terms of the primary efficacy outcome (composite of any DVT, nonfatal PE, and all-cause mortality), without significant differences in the rates of major bleeding [47–50]. 

Huisman et al. performed a pooled analysis of the RECORD1, RECORD3, and RECORD4 studies and found that the risk of symptomatic VTE plus all-cause mortality among patients treated with enoxaparin was 2-fold higher than that among patients treated with rivaroxaban (1.2% versus 0.6%; odds ratio, 2.04; 95% CI, 1.32 to 3.17; *P* < 0.001) [30]. The composite of major and clinically relevant nonmajor bleeding was significantly lower in patients treated with enoxaparin versus rivaroxaban (2.5% versus 3.1%; odds ratio, 0.79; 95% CI, 0.62–0.99; *P* = 0.049). Turpie et al. also performed a pooled analysis of the four RECORD studies [51] and concluded that the composite risk of symptomatic VTE and all-cause mortality after elective THA or TKA in patients treated with rivaroxaban was significantly lower than in patients treated with enoxaparin. These findings were consistent across patient subgroups, irrespective of age, sex, body weight, or creatinine clearance. The rate of bleeding in patients receiving rivaroxaban was slightly higher than that in patients receiving enoxaparin; however, fewer serious adverse events were observed in patients receiving rivaroxaban than in patients receiving enoxaparin [51]. Performing separate meta-analyses of dabigatran and rivaroxaban and comparing the results provided an indirect comparison of the efficacy and safety profiles of these agents in the prevention of VTE, which suggested that dabigatran and rivaroxaban might not differ in efficacy or safety profile outcomes in the prevention of VTE [52].

#### 3.1.3. Primary VTE Prevention in Hospitalized Acutely Ill Medical Patients

The MAGELLAN trial was a randomized, parallel-group efficacy and safety study of rivaroxaban for the prevention of VTE in hospitalized acutely ill medical patients [53]. The study compared oral rivaroxaban (10 mg once daily for 35 ± 4 days) with subcutaneous enoxaparin (40 mg once daily for 10 ± 4 days) followed by placebo and found that rivaroxaban was noninferior in reducing the risk of VTE at day 10. At day 35, extended thromboprophylaxis with rivaroxaban was superior to enoxaparin followed by placebo in reducing the risk of VTE. Overall rates of clinically relevant bleeding were low in both arms, but bleeding was significantly higher in the rivaroxaban arm across the entire study period. Rates of other adverse events, including liver and cardiovascular events, and all-cause mortality were similar in both arms.

#### 3.1.4. Treatment and Secondary Prevention of VTE

Three randomized phase III clinical trials, the EINSTEIN-DVT, EINSTEIN-PE, and EINSTEIN-Extension studies, evaluated the efficacy and safety of rivaroxaban in the setting of the treatment and secondary prevention of VTE. In the EINSTEIN-DVT study, rivaroxaban (15 mg twice daily for 3 weeks followed by 20 mg daily) was compared with enoxaparin followed by a vitamin K antagonist, for 3–12 months, in patients with acute symptomatic DVT (without PE) [54]. Rivaroxaban was found to have noninferior efficacy in preventing symptomatic recurrent DVT (2.1% versus 3.0%; hazard ratio 0.68; *P* < 0.001) with a similar safety profile. Using a similar treatment approach, the recently completed EINSTEIN-PE trial compared rivaroxaban with standard therapy (enoxaparin followed by a vitamin K antagonist) for 3–12 months in patients with acute symptomatic PE (with or without DVT) [55]. The results suggested that rivaroxaban is noninferior to standard therapy (noninferiority margin, 2.0; *P* = 0.003) for preventing symptomatic recurrent VTE, with major bleeding rates significantly higher in the standard-therapy group. In the EINSTEIN-Extension study, an additional 6–12-month course of rivaroxaban (20 mg once daily) was compared with placebo in patients who had completed 6–12 months of anticoagulant treatment for VTE [56]. As expected, rivaroxaban had superior efficacy compared to placebo with 82% relative risk reduction in the recurrence of VTE. The rate of clinically relevant nonmajor bleeding was higher in the rivaroxaban group (5.4% versus 1.2%).

#### 3.1.5. Practical Information

In the United States, Canada, and Europe, rivaroxaban is approved for the prevention of VTE in patients who have undergone elective THR or TKR surgery. In the United States, rivaroxaban is approved to treat DVT or PE, and to reduce the risk of recurrent DVT and PE following initial treatment, whereas in Europe and Canada it is indicated for the treatment of DVT and secondary prevention of VTE (Table 4) [42, 57, 58].

The recommended doses of rivaroxaban for VTE prevention in patients following THR or TKR are 10 mg orally once daily for 35 days and 10 mg orally once daily for 12–14 days, respectively [42, 57, 58]. The initial dose should be taken at least 6–10 hours after surgery once hemostasis has been established. When rivaroxaban is used to treat DVT or to prevent recurrent DVT and PE, the recommended dose for the initial treatment of acute DVT is 15 mg twice daily for the first 3 weeks followed by 20 mg once daily [57]. 

Rivaroxaban is contraindicated in patients with moderate (Child-Pugh B) or severe (Child-Pugh C) hepatic impairment, patients with any hepatic disease associated with coagulopathy, and patients with severe renal impairment (CrCl < 30 mL/min) [42]. Patients who develop acute renal failure while on rivaroxaban should discontinue the treatment. It is not recommended to use rivaroxaban concomitantly with combined P-gp and strong CYP3A4 inhibitors (e.g., ketoconazole, itraconazole, lopinavir, ritonavir, indinavir, and conivaptan) or with combined P-gp and strong CYP3A4 inducers (e.g., carbamazepine, phenytoin, rifampin, and St. John's wort) [42]. If anticoagulation must be discontinued to reduce the risk of bleeding from surgery or other procedures, rivaroxaban should be stopped at least 24 hours before the procedure. An epidural catheter should not be removed earlier than 18 hours after the last administration of rivaroxaban. The next rivaroxaban dose should not be administered earlier than 6 hours after the removal of the catheter [42]. If traumatic puncture occurs during epidural catheter insertion, the administration of rivaroxaban should be delayed for 24 hours [42].

### 3.2. Apixaban

#### 3.2.1. Pharmacology

Apixaban is a potent, orally active inhibitor of free and clot-bound coagulation factor Xa [59–62]. Apixaban binds directly to the active site of factor Xa and exerts anticoagulant effects by inhibiting the conversion of prothrombin to thrombin. In addition, it inhibits trypsin and thrombin generation. Apixaban has an oral bioavailability of approximately 50% and a high protein binding percentage of 87% [62]. It reaches peak concentrations 3-4 hours after administration and has a half-life of 8–15 hours [62, 63]. Apixaban is predominantly eliminated through the cytochrome P450 CYP3A4/5-related pathway. Twenty-seven percent of the drug is excreted renally [62]. Apixaban is a substrate of transport proteins, P-gp, and breast cancer resistance protein [62].

#### 3.2.2. Primary VTE Prevention in Patients after Major Orthopedic Surgery

Apixaban was evaluated in three phase III clinical trials; the ADVANCE-1 and ADVANCE-2 trials which evaluated the use of apixaban following TKR for 10–14 days, and the ADVANCE-3 trial which evaluated the use of apixaban following THR for 35 days. In the ADVANCE-1 trial, apixaban (2.5 mg twice daily for 10–14 days) was compared with enoxaparin (30 mg twice daily for 10–14 days). Apixaban did not meet the prespecified statistical criteria for noninferiority in terms of efficacy (composite of any DVT, nonfatal PE, and all-cause mortality), but its use was associated with lower rates of clinically relevant bleeding [64]. However, in both the ADVANCE-2 and ADVANCE-3 trials, apixaban (2.5 mg twice daily) showed superior efficacy compared to enoxaparin (40 mg daily) [65, 66]. There were no significant differences in the rates of major bleeding between apixaban and enoxaparin [64, 66]. 

Huang et al. performed a meta-analysis of the ADVANCE-1 and ADVANCE-2 trials and a phase II study of apixaban in patients undergoing TKR surgery and concluded that apixaban is noninferior to subcutaneous enoxaparin when used for the same duration and has a considerable advantage regarding the safety profile of major bleeding [67].

#### 3.2.3. Primary VTE Prevention in Hospitalized Acutely Ill Medical Patients

The ADOPT study was a double-blind, double-dummy, placebo-controlled trial that compared extended-duration apixaban (2.5 mg twice daily for 30 days) with standard-duration enoxaparin (40 mg daily for 6–14 days) [68]. This study indicated that, in medically ill patients, an extended course of thromboprophylaxis with apixaban was not superior to a shorter course with enoxaparin. Apixaban was associated with significantly more major bleeding events than enoxaparin.

#### 3.2.4. Primary VTE Prevention in Patients with Metastatic Cancer Undergoing Chemotherapy

Levine et al. performed a randomized, double-blind phase II dose-ranging study investigating the efficacy and safety of apixaban versus placebo in patients with metastatic cancer undergoing chemotherapy [69]. The treatment duration was 12 weeks. The authors concluded that apixaban was well tolerated in their study population, and supported pursuing phase III clinical trials of apixaban in preventing VTE in cancer patients receiving chemotherapy. However, it should be noted that the study protocol was potentially selected for patients at low risk of bleeding (e.g., exclusion of patients receiving antiplatelets or bevacizumab or those with prolonged coagulation times) and the sample size is small.

#### 3.2.5. Treatment and Secondary Prevention of VTE

The BOTTICELLI trial was a phase II dose-ranging study assessing the efficacy and safety of apixaban versus standard therapy (low molecular weight heparin followed by a vitamin K antagonist) in the treatment of patients with acute symptomatic DVT for 84–91 days [70]. The study concluded that a fixed dose of apixaban may be given as the sole treatment for DVT. Two ongoing phase III clinical trials, the AMPLIFY (NCT00643201) and AMPLIFY-EXT (NCT00633893) trials, evaluate the efficacy and safety of apixaban for the standard duration and extended duration treatment of DVT or PE, respectively. 

#### 3.2.6. Practical Information

Apixaban is currently approved in Canada and Europe for the prevention of VTE in adults following THR or TKR [62, 71]. In both these patient groups, apixaban (2.5 mg orally twice daily) should be started 12–24 hours after the operation and then continued daily for 10–14 days in TKR patients or for 32–38 days in THR patients [62, 71]. Apixaban is contraindicated in patients who have liver disease associated with coagulopathy and a clinically relevant bleeding risk, and in patients who are undergoing concomitant systemic treatment with strong inhibitors of both cytochrome P450 CYP3A4 and P-gp [71].

### 3.3. Edoxaban

#### 3.3.1. Pharmacology

Edoxaban is an orally active, competitive, direct inhibitor of factor Xa that is currently undergoing phase III clinical trials for the prevention of stroke in patients with atrial fibrillation and for the prevention and treatment of VTE. Edoxaban has more than 10000-fold greater selectivity for factor Xa than thrombin [72]. With a time to peak plasma concentration of 1-2 hours, the anticoagulant effect of edoxaban has a rapid onset and is sustained for up to 24 hours [73]. Similar to apixaban, edoxaban has absolute oral bioavailability of approximately 50% and a half-life of 9–11 hours in young healthy subjects. Thirty-five percent of the drug is renally excreted (24% as active metabolite) and 62% is excreted via feces [74]. The metabolism in liver microsomes is mediated mainly by CYP3A4-related pathways. Because edoxaban is a substrate of P-gp, strong inhibitors of P-gp could lead to a higher concentration of the drug [75]. Edoxaban can be administered with or without food [76]. 

#### 3.3.2. Primary VTE Prevention in Patients after Major Orthopedic Surgery

A randomized, double-blind, placebo-controlled, dose-ranging phase IIb trial, which was conducted to evaluate the efficacy and safety of edoxaban for the prevention of VTE in Japanese patients undergoing TKR, revealed that edoxaban demonstrated significant dose-dependent reductions in VTE in patients undergoing TKR and had a bleeding incidence similar to that of placebo [73]. Another randomized, double-blind, dose-response phase II trial of edoxaban in patients undergoing elective THR also showed that edoxaban had a significant dose-response relationship for efficacy across the once-daily dose groups in preventing VTE, and a low bleeding incidence similar to dalteparin [77].

A pooled analysis of two phase III clinical trials in the Japanese population, the STARS E-3 trial, which investigated edoxaban following TKR and STARS J-5 trial, which investigated edoxaban following THR revealed that the rate of VTE events among patients receiving edoxaban (30 mg once daily) was significantly lower than that among patients receiving enoxaparin (20 mg twice daily) (5.1% versus 10.7%, *P* < 0.001) and that the groups' rates of adverse events did not differ significantly (4.6% versus 3.7%, *P* = 0.427) [78]. Further clinical investigation of the efficacy and safety of once-daily edoxaban in comparison with low-molecular weight heparin/warfarin in the treatment and prevention of VTE in patients with acute DVT and/or PE is being conducted in the HOKUSAI-VTE phase III trial (NCT00986154). 

#### 3.3.3. Practical Information

Edoxaban is currently approved in Japan for the prevention of VTE after major orthopedic surgery [79]. However, because enoxaparin 20 mg twice daily is not a popular dosing regimen outside Japan, it may not be appropriate to extrapolate the results of the STARS trials to other patient populations. Further phase III studies in wider population are required before the drug can be extensively approved for use in the prevention of VTE. 

## 4. Monitoring the Anticoagulant Effect

All new oral anticoagulants have a predictive dose response, which permits standard dosing without the need for routine monitoring. All oral factor Xa inhibitors cause a dose-dependent increase in aPTT, PT, INR, and one-step prothrombinase-induced clotting time, especially at supratherapeutic doses [80–82]. Antifactor Xa activity was suggested to be a better indicator of plasma concentration of oral factor Xa inhibitors than aPTT, PT, or INR [80, 82]. In contrast, dabigatran significantly alters aPTT, ECT, TT, and, to a lesser extent, PT and INR values at therapeutic doses [23]. Measurement of TT or ECT may be used to evaluate the anticoagulant effect of dabigatran in patients who develop bleeding complications [83]. New tests are currently being developed to enable the exact quantification of the anticoagulant effect of oral direct factor Xa inhibitor and of dabigatran therapy by means of chromogenic Factor Xa assays [84] and by specific tests such as HemoClot thrombin inhibitor assay [85], respectively.

## 5. Management of Bleeding Complications

Bleeding complications are the main concern in patients receiving anticoagulant therapy. Currently there is no specific antidote to reverse the effects of new oral anticoagulants. In cases of mild to moderate bleeding, routine management involving stoppage of the inciting oral anticoagulant, mechanical compression, surgical, endoscopic, or interventional therapy, and hemodynamic stabilization will suffice. If severe bleeding occurs, one should consider using fresh frozen plasma, prothrombin complex concentrates, recombinant factor VIIa, or factor eight inhibitor bypassing activator [83, 86]. In patients receiving dabigatran, hemodialysis can be used to reduce the drug level [83]. In case of drug overdose, activated charcoal may be given within 3 hours of oral anticoagulant intake to reduce gastrointestinal absorption. Antibodies capable of neutralizing dabigatran are being developed [87].

## 6. New Oral Anticoagulants in the Pipeline

Other newer oral anticoagulants that are currently under evaluation in clinical trials include a direct thrombin inhibitor, AZD0837 (AstraZeneca, phase II), and several direct factor Xa inhibitors, betrixaban (Portola Pharmaceuticals, phase III), eribaxaban (Pfizer, phase II), letaxaban (Takeda Pharmaceuticals, phase II), and LY-517717 (Lilly, phase II) [88].

## 7. Summary

Although the half-lives of the new oral anticoagulants vary, they all reach maximum concentrations within approximately 1–4 hours. Rivaroxaban and edoxaban offer the benefit of single daily dosing. The benefits and limitations of the new oral anticoagulants are summarized in Table 3, and their approved indications for use in different countries are given in Table 4. 

Dabigatran etexilate (150 mg or 220 mg daily; RE-MOBILIZE study) and apixaban (2.5 mg twice daily; ADVANCE-1 study) failed to show noninferiority compared to enoxaparin (30 mg twice daily) in preventing VTE in patients after TKR [25, 64]. However, the RECORD4 trial revealed that (rivaroxaban 10 mg once daily) conferred superior efficacy in a similar clinical setting without significant increase in major adverse events [50]. Moreover, rivaroxaban was found to be less costly and more effective than dabigatran or enoxaparin for use in thromboprophylaxis in patients after TKR or THR in a cost-effectiveness analysis [89]. These findings may be in favor of rivaroxaban as the preferred prophylactic antithrombotic agent in patients undergoing hip or knee replacement. In contrast, studies of edoxaban in the non-Japanese population should be done to establish wider clinical applicability in a similar setting. 

In the treatment and secondary prevention of VTE, both dabigatran etexilate twice daily and rivaroxaban once daily exhibited noninferior efficacy compared to warfarin when given for 3–12 months [32, 54, 55]. However, the REMEDY trial revealed that dabigatran was less effective and associated with more acute coronary events compared to warfarin when given for an extended period of up to 36 months for the prevention of recurrent VTE [34]. There was no similar study done with rivaroxaban. Two phase III clinical trials, the AMPLIFY (NCT00643201) and AMPLIFY-EXT (NCT00633893) trials, are underway to evaluate the efficacy and safety of apixaban for the treatment and secondary prevention of VTE. Similarly, a phase III multinational study of edoxaban is currently under way (NCT00986154).

 Extended thromboprophylaxis with rivaroxaban (as per the MAGELLAN study findings) [53], but not apixaban (as per the ADOPT study findings) [68], was found to be superior to enoxaparin followed by placebo for primary VTE prevention in hospitalized acutely ill medical patients. Both rivaroxaban and apixaban were associated with significantly more bleeding complications than enoxaparin. Thus, current evidence does not support the routine use of the new oral anticoagulants for thromboprophylaxis in hospitalized medical patients.

## Figures and Tables

**Table 1:** Limitations of traditional anticoagulants.

Warfarin	
Narrow therapeutic window	
Unpredictable pharmacokinetic and pharmacodynamic properties	
Significant interaction with food and drugs	
Slow onset and offset of action	
Need for regular anticoagulation monitoring and dose adjustment	
High incidence of intracranial bleeding, especially among Asian patients	
UFH/LMWH	
Parenteral administration only	
Risk of thrombocytopenia	
Need for laboratory monitoring (platelet count)	

UFH: unfractionated heparin, LMWH: low molecular weight heparin.

**Table 2:** Pharmacologic profiles of new oral anticoagulants in clinical use.

	Dabigatran	Rivaroxaban	Apixaban	Edoxaban
Target	Thrombin	Factor Xa	Xa	Xa
Administration	Once or twice daily	Once daily	Twice daily	Once daily
Prodrug	Yes	No	No	No
Half-life (hours)	12–17	5–9	8–15	9–11
*t*_max⁡_ (hours)	0.5–2	2–4	3-4	1-2
Bioavailability	3–7%	66–100%	50%	50%
Protein binding	35%	92–95%	87%	40–59%
Renal excretion	80%	33%	27%	35%

*t*_max⁡_: time to peak.

**Table 3:** Benefits and limitations of the new oral anticoagulants.

Benefits	
Rapid onset and offset of action	
Relative ease of oral administration	
No need for routine monitoring of anticoagulant effect	
Lack of significant drug interactions	
Limitations	
No antidote	

**Table 4:** Approved indications of new oral anticoagulants in USA, Canada, and Europe.

	Dabigatran	Rivaroxaban	Apixaban	Edoxaban
Prevention of venous thromboembolism after major orthopedic surgery	Canada, Europe	USA, Canada, and Europe	Canada, Europe	Japan
Prevention of systemic thromboembolism in nonvalvular atrial fibrillation	USA, Canada	USA, Canada, and Europe	USA, Canada, and Europe	NA
Treatment of acute DVT/PE and prevention of recurrent VTE	NA	USA, Canada*, and Europe*	NA	NA

DVT: deep venous thrombosis, NA: not applicable, PE: pulmonary embolism.

*Only for treatment of acute DVT and prevention of recurrent VTE.
